# Pyrroline-5-Carboxylate Reductase-2 Promotes Colorectal Carcinogenesis by Modulating Microtubule-Associated Serine/Threonine Kinase-like/Wnt/β-Catenin Signaling

**DOI:** 10.3390/cells12141883

**Published:** 2023-07-18

**Authors:** Raju Lama Tamang, Balawant Kumar, Sagar M. Patel, Ishwor Thapa, Alshomrani Ahmad, Vikas Kumar, Rizwan Ahmad, Donald F. Becker, Dundy (Kiran) Bastola, Punita Dhawan, Amar B. Singh

**Affiliations:** 1 Department of Biochemistry and Molecular Biology, University of Nebraska Medical Center, 985870 Nebraska Medical Center, Omaha, NE 68198-6125, USA; 2 Department of Biochemistry and Redox Biology Center, University of Nebraska-Lincoln, Lincoln, NE 68588, USA; 3 School of Interdisciplinary Informatics, College of Information Science & Technology, University of Nebraska at Omaha, Omaha, NE 68182, USA; 4 Department of Pathology and Microbiology, University of Nebraska Medical Center, 985870 Nebraska Medical Center, Omaha, NE 68198-6125, USA; 5 Department of Genetics, Cell Biology, and Anatomy, University of Nebraska Medical Center, 985870 Nebraska Medical Center, Omaha, NE 68198-6125, USA; 6 Veterans Affairs Nebraska-Western Iowa Health Care System, Omaha, NE 68105-1850, USA; 7 Fred and Pamela Buffett Cancer Center, University of Nebraska Medical Center, Omaha, NE 68198-65870, USA

**Keywords:** pyrroline 5 carboxylate reductases (PYCRs), colorectal cancer (CRC), proline metabolism, cancer progression, MASTL, Wnt signaling, proteomics

## Abstract

Background: Despite significant progress in clinical management, colorectal cancer (CRC) remains the third most common cause of cancer-related deaths. A positive association between PYCR2 (pyrroline-5-carboxylate reductase-2), a terminal enzyme of proline metabolism, and CRC aggressiveness was recently reported. However, how PYCR2 promotes colon carcinogenesis remains ill understood. Methods: A comprehensive analysis was performed using publicly available cancer databases and CRC patient cohorts. Proteomics and biochemical evaluations were performed along with genetic manipulations and in vivo tumor growth assays to gain a mechanistic understanding. Results: PYCR2 expression was significantly upregulated in CRC and associated with poor patient survival, specifically among PYCR isoforms (PYCR1, 2, and 3). The genetic inhibition of PYCR2 inhibited the tumorigenic abilities of CRC cells and in vivo tumor growth. Coinciding with these observations was a significant decrease in cellular proline content. PYCR2 overexpression promoted the tumorigenic abilities of CRC cells. Proteomics (LC-MS/MS) analysis further demonstrated that PYCR2 loss of expression in CRC cells inhibits survival and cell cycle pathways. A subsequent biochemical analysis supported the causal role of PYCR2 in regulating CRC cell survival and the cell cycle, potentially by regulating the expression of MASTL, a cell-cycle-regulating protein upregulated in CRC. Further studies revealed that PYCR2 regulates Wnt/β-catenin-signaling in manners dependent on the expression of MASTL and the cancer stem cell niche. Conclusions: PYCR2 promotes MASTL/Wnt/β-catenin signaling that, in turn, promotes cancer stem cell populations and, thus, colon carcinogenesis. Taken together, our data highlight the significance of PYCR2 as a novel therapeutic target for effectively treating aggressive colon cancer.

## 1. Introduction

Colorectal cancer (CRC) is the second most common cause of cancer and the third leading cause of cancer-related death, in both males and females combined [1]. Despite significant advances in technology and clinical management, the overall patient survival of CRC patients remains meager, especially when the cancer metastasizes to distant organs [2]. Cancer stem cells (CSCs) promote cancer malignancy and metastasis [3]. However, mechanisms that cause CSCs to foster CRC are still not well understood and thus require further investigation. In this regard, the ability of CSCs to harness cell metabolic pathways for their survival is an area of intense research [4]. Studies have supported the key role of proline metabolism in promoting oncogenic growth [5]. However, the complexity of the molecular apparatus that regulates proline metabolism has hindered our understanding of the specific roles of proteins involved in oncogenic growth and progression and, thus, therapeutic utility. 

The terminal reaction in the proline metabolic pathway is the reduction of Δ^1^-pyrroline-5-carboxylate (P5C) into proline, which is catalyzed by P5C reductase (PYCR). Human PYCR has three known isoforms, PYCR1, PYCR2, and PYCRL/PYCR3 [6]. The generation of P5C takes place via distinct metabolic routes such as from the glutamate pathway or the ornithine pathway, and accordingly, the PYCR isoforms have distinct cellular localizations [7,8]. PYCR1 and PYCR2 are localized on the inner mitochondrial membrane, but a recent study showed that PYCR2 may also be present in the cytosol [9]. PYCRL/PYCR3 is found in the cytosol [9,10]. P5C generated by the glutamate pathway appears to be preferred by PYCR2 for proline biogenesis, which utilizes NADH as the reducing substrate.

Among the PYCR isoforms, PYCR1 has been studied the most extensively for its role in carcinogenesis, including in cancers of the kidney, lung, and prostate [11,12,13]. Also, PYCRL/PYCR3 regulates metabolic reprogramming in cancer cells [14]. PYCR2 remains the least investigated enzyme for its role in cancer, though recent studies have demonstrated its prognostic significance in cervical; hepatocellular; and, recently, colon carcinoma [15,16]. A non-cancer role for PYCR2 in brain abnormalities and neuronal dysfunctions such as hypomyelination, microcephaly, leukodystrophy, and spastic paraplegia has also been reported [17,18,19].

The current study was undertaken to investigate the role of PYCR2 in colon carcinogenesis as there were no reports on its role/regulation in CRC at the time of the inception of the current study. Based on our comprehensive investigation and biochemical evidence, we report here that PYCR2 is significantly upregulated in CRC. We further show that, in addition to modulating the proline metabolism of CRC cells, PYCR2 modulates the CSC niche, potentially by altering microtubule-associated serine/threonine kinase-like (MASTL)/Wnt/β-catenin signaling. Overall, our data support the novel role of PYCR2 in regulating MASTL/Wnt signaling to promote CRC.

## 2. Materials and Methods

### 2.1. Bioinformatic Assessment of the Public Databases

An assessment of a public database was performed on 22 November 2021 as described previously [20]. In brief, a web-based public resource for cancer OMICS studies, UALCAN (The University of Alabama at Birmingham Cancer Data Analysis Portal: (http://ualcan.path.uab.edu/), was used to examine the TCGA (The Cancer Genome Atlas Program) database. The CPTAC (Clinical Proteomic Tumor Analysis Consortium) database was analyzed for PYCR2 protein expression. A Kaplan–Meier survival analysis was performed to examine the association of PYCR expression with patient survival [21]. Similarly, relevant public databases were analyzed to determine the relative expression of PYCR2 in normal and primary tumors among French (GSE39582), Amsterdam (GSE33113), and Korean colon cancer populations [22].

### 2.2. Cell Culture

CRC cell lines HCT116, SW620, SW480, HT29, DKO1, and Caco2 were purchased from ATCC or were already available in our laboratory. IEC-6 cells were used as normal colon epithelial cells. HCT116, SW620, SW480, and HT29 cell lines were cultured in Roswell Park Memorial Institute Medium-1640 (RPMI-1640) culture media, while Dulbecco’s Modified Eagle’s Medium (DMEM) was used to maintain other cell lines, such as DKO-1, IEC-6, and Caco2 cells. The culture medium was supplemented with 10% FBS and a 1% Penicillin/Streptomycin antibiotic cocktail. The cells were cultured in humidified incubator supplied with 5% CO_2_ unless mentioned otherwise.

### 2.3. Loss and Gain of Expression and Function in PYCR2 and MASTL

To determine the causal role of PYCR2, its expression was inhibited using several genetic tools, including siRNA and shRNA plasmids or CRISPR-Cas9-mediated gene knockout. Anti-human PYCR2 siRNA was purchased from Ambion, Carlsbad, CA, USA. Anti-human PYCR2 doxycycline-inducible shRNA plasmid with GFP tag (Dharmacon, Lafayette, CO, USA) and anti-human *PYCR2* shRNA plasmid construct (Sigma Aldrich, St. Louis, MO, USA) were used. Turbofectin (Turbo DNAfectin^TM^ 3000, Bulls Eye, Valley Park, MO, USA) was used for transfections as per manufacturer protocol. Transfected cells were selected to stabilize the expression of shRNA (inducible and constitutive). The cells transfected with constitutive shRNA plasmid were selected using puromycin to generate stably transfected PYCR2 knockdown (KD) cell lines (HCT116 cells; 5.5 μg/mL and SW620 cells; 5.0 μg/mL). For doxycycline-inducible shRNA expression, cells received 1 μg/mL of freshly prepared doxycycline/day for 72 h. CRISPR/Cas9 sgRNA with a mCherry tag for knocking out PYCR2 expression was purchased from Genecopia, Rockville, MD, USA. After transfection with CRISPR/Cas9 sgRNA, selection was performed by using G418 500 µg/mL to generate a stable HCT116 PYCR2 knockout (KO) cell.

The PYCR2 overexpression plasmid was constructed in our laboratory, where full-length PYCR2 cDNA was cloned in a pcDNA3 plasmid construct under a CMV promoter. The PYCR2 insertion was confirmed via DNA sequencing, and resultant protein expression was confirmed using immunoblotting. The SW480 cells were used for stable transfection, and selection was performed using 400 μg/mL of G418. To overexpress MASTL in PYCR2-KO HCT116 cells, we used the pCMVSPORT6_MASTL expression construct (Transomic, Huntsville, AL, USA). To inhibit MASTL expression/activity in PYCR2-overexpressing CRC cells, we used a known inhibitor of MASTL (Great Wall Kinase inhibitor (GKI); 25 μM)). The details of the siRNA and plasmids used in the study are provided in Supplementary Table S1.

### 2.4. Cell Viability Assay

Cell viability assays were performed as described previously [23]. In brief, 5 × 10^3^ cells/well were cultured in a 96-well culture plate. The following day, cells were treated with Presto blue reagent (1:10 ratio) for 10 min followed by incubation at 37 °C and subjected to fluorescence measurement (560 nm (excitation) and 590 nm (emission)).

### 2.5. Cell Invasion Assay

Cell invasion assays were performed using transwell filters as described previously [23]. In brief, 5 × 10^4^ cells/well were grown in a culture medium without FBS on top of the transwell inserts. The lower chamber of the well was filled with the complete media. The amount of cell invasion was determined after 72 h.

### 2.6. Wound-Healing Assay

Wound-healing assays were performed as described previously [23]. The cell migration was determined by analyzing the wound healing area every 24 h post-wounding. The infinity analyzer software was used for the measurement of the area of the wounds at the respective times of study.

### 2.7. Soft Agar Assay

These assays were performed to determine the anchorage-independent cell growth, as described previously [23].

### 2.8. Sphere Forming Assay

Sphere-forming assays were conducted as described previously [24,25]. Low attachment plates were used to examine the sphere-forming ability in the control and PYCR2-manipulated CRC cells. Cell density of 5 × 10^3^ was used for initial plating in spheroid-specific culture medium (Supplementary Table S2), and sphere growth was analyzed every 24 h for the next 8–10 days.

### 2.9. Immunohistochemical (IHC) Analysis

All IHC analyses were performed as described before [23]. In brief, Tris EDTA buffer (pH 9.0) or sodium citrate buffer (pH 6.0) was used for epitope unmasking using a pressurizing chamber. The primary antibody was incubated with the tissue section of interest for overnight incubation at 4 °C. The secondary biotinylated antibody (ABC polymer kit) was incubated for 45 min, and color development was performed using DAB (3’3’diaminobenzidine). Analysis of the PYCR2 staining and intensity scoring was performed by a pathologist in a blinded manner. The analysis was based on scoring the intensity score of the PYCR2 immunostaining as 0, +1, +2, or +3. The mean of the intensity score of the PYCR2 staining was plotted. The details of the antibodies and reagents used for the study are provided in Supplementary Table S2.

### 2.10. Immunofluorescence (IF) Analysis

The IF analysis was performed as described previously [26]. Images were captured using a Nikon Eclipse Ti inverted microscope. The NIS Elements BR 4.30.01 (64 bit) software was used for the analysis of the IF images.

### 2.11. Fluorescence-Activated Cell Sorting (FACS) Analysis for Determining Apoptosis and Cell Cycle Progression

An apoptosis kit was purchased from BD Bioscience, San Jose, CA, USA. The standard manufacturer protocol was followed. A single-cell suspension was prepared and stained with Annexin-V-FITC and propidium iodide (PI) before a FACS analysis. Cell cycle analysis was performed as described previously [27]. In brief, a single-cell suspension was prepared. The cell suspension was incubated with RNAse-A (1 mg/mL) followed by 5 µL of PI (1 mg/mL) at room temperature (in dark) before the cell cycle analysis.

### 2.12. Western Blot Analysis

Western blots were performed as described previously [23]. Information about the reagents and dilutions used for the analysis is provided in Supplementary Table S2. The development of blots and densitometric analysis were performed using Image Lab (5.0), a Bio-Rad program.

### 2.13. RT-qPCR Analysis

The primers for *PYCR1*, *PYCR2*, *PYCR3*, *CD133*, *SOX2*, and *ACTB* (β-actin) were purchased from Integrated DNA Technologies (IDT), Coralville, IA, USA, and details are provided in Supplementary Table S3. RT-qPCR was performed as described before [28]. β-actin was used as a housekeeping gene to normalize the data, and statistical analysis was performed using GraphPad Prism 9.5.0.

### 2.14. Xenograft Tumor Growth Assay

Athymic/nude mice (6–8 weeks old) were used for an in vivo xenograft study. Both studies were performed under an approved protocol by the Institutional Animal Care and Use Committee (IACUC:17-126-11FC). For the in vivo xenograft studies, SW620 cells were injected into nude mice (*n* = 3/group). Exponentially growing control and PYCR2-KD cells were trypsinized, and single-cell suspensions (1 × 10^6^ cells/100 µL) were injected into the lower flanks of the nude mice [23]. The left flank was used for the injection of control cells, while the right flank was used for the injection of PYCR2-KD cells. The body weight and tumor dimensions were measured every second day. The tumor volume was calculated using the following formula: tumor volume = width^2^ × length × 0.5 mm^3^. The mice were sacrificed on the 20th day following the injection of cancer cells, and tumors were then isolated from the flanks, and the tumor weight was recorded.

### 2.15. Colonoscopy-Guided Intramucosal Transplantation of CRC Cells for Tumor Development

Colonoscopy-guided injection of CRC cells into the colonic mucosa was performed as previously described by our laboratory [29]. Control and PYCR2-KO HCT116 cells (10^6^ cells/50 µL) were used for intramucosal transplantation into the colon walls of nude mice (*n* = 4/group) by using a small-animal colonoscope (COLOVIEW; Karl-Storz, Tuttlingen, Germany). Mice were longitudinally monitored using a colonoscope to determine the colon tumor growth in mice receiving both control and PYCR2-KO CRC cells. Mice were sacrificed on the 33rd day following the injection of the respective cancer cells, and the status of colon tumor growth was determined.

### 2.16. Proteomics (LC-MS/MS) Analysis

The total protein lysate (50 µg/100 μL sample) from five biological replicates from each group was used. Detergent was removed via chloroform/methanol extraction, and a protein pellet was resuspended in 100 mM ammonium bicarbonate and digested with MS-grade trypsin (Pierce Biotechnology, Waltham, MA, USA) overnight at 37 °C following reduction with 10 mM DTT at 56 °C for 30 min and alkylation using 50 mM iodoacetamide at RT for 25 min.

Peptides were cleaned with PepClean C18 spin columns (ThermoFisher Scientific, Waltham, MA, USA) and were resuspended in 2% acetonitrile (can) and 0.1% formic acid (FA). In total, 500 ng of each sample was loaded onto trap columns (Acclaim PepMap 100 75 µm × 2 cm C18 liquid chromatography (LC) columns (Thermo Scientific™, Waltham, MA, USA) at a flow rate of 4 µL/min; then, the samples were separated with a Thermo RSLC Ultimate 3000 (Thermo Scientific™) on a Thermo Easy-Spray PepMap RSLC C18 75 µm × 50 cm C-18 2 µm column (Thermo Scientific™) with a step gradient of 4–25% solvent B (0.1% FA in 80% ACN) from 10–100 min and 25–45% solvent B for 100–130 min at 300 nl/min and 50 °C with a 155 min total run time.

The eluted peptides were analyzed using a Thermo Orbitrap Fusion Lumos Tribrid (Thermo Scientific™) mass spectrometer in a data-dependent acquisition mode. A survey full-scan MS (from *m/z* 350–1800) was acquired in the Orbitrap with a resolution of 120,000. The AGC target for MS1 was set as 4 × 10^5^, and the ion-filling time was set as 100 ms. The most intense ions with a charge state of 2–6 were isolated in a 3 s cycle, fragmented using HCD fragmentation with 35% normalized collision energy, and detected at a mass resolution of 30,000 at 200 *m/z*. The automatic gain control (AGC) target for MS/MS was set as 5 × 10^4^, and the ion-filling time was set for 60 ms; dynamic exclusion was set for 30 s with a 10 ppm mass window. Each sample was run in duplicates. Protein identification was performed by searching for MS/MS data in the Swissport human protein database downloaded on September 2021 using the in-house PEAKS X + DB search engine. The search was set up for full tryptic peptides with a maximum of two missed cleavage sites. The acetylation of protein N-terminus and oxidized methionine were included as variable modifications, and the carbamidomethylation of cysteine was set as a fixed modification. The precursor mass tolerance threshold was set as 10 ppm, and the maximum fragment mass error was 0.02 Da. The significance threshold of the ion score was calculated based on a false discovery rate of ≤1%. Quantitative data analysis was performed using Progenesis QI for proteomics 4.2 (Nonlinear Dynamics). Statistical analysis was performed using ANOVA, and the Benjamin–Hochberg (BH) method was used to adjust *p*-values for multiple testing-caused false discovery rates. An adjusted *p* of ≤0.05 was considered significant. Various plots, such as heatmap, volcano plot, and PCA, were generated using Partek Genomics Suite 7.0.

Consensus path DB by Max Planck Institute for Molecular Genetics [http://cpdb.molgen.mpg.de/, released 35(05.06.2021)] was utilized for KEGG pathway analysis and GO biological function in the control and experimental groups.

### 2.17. Intracellular Proline Measurement

Control and PYCR2-KO HCT116 cells were cultured separately in DMEM and RPMI-1640 for 24 h. Biological triplicates were made from each sample. Ice-cold 100% or 80% methanol was utilized for cell sample quenching as well as cell lysis, as per a recent metabolomics study [30]. The measurement of the intracellular L-Proline concentration was performed using a modified acid ninhydrin assay, as described previously [31,32]. Fresh ninhydrin was dissolved at 1.5 mg/mL in glacial acetic acid before each assay. L-Proline known standards (0–500 μM) were prepared in methanol solvent, and 15 µL of a standard sample was sequentially mixed with 15 µL 3 M Na-acetate buffer and 200 µL ninhydrin solution (to achieve a final pH of 3.0 altogether) in a 96-well microplate. The samples were immediately analyzed in a 96-well microplate reader spectrophotometer (Synergy 2, BioTek Intsruments, Inc., Winooski, VT, USA) at 352 nm (in sweep mode, endpoint absorbance reading) as an initial time-zero measurement. Then, the microplate was covered in foil to protect samples from light and subjected to 50 °C static incubation for 12.5 min followed by cooling down to room temperature for 1 h. Lastly, a final absorbance measurement was taken in the plate reader at 352 nm. The difference in the A_352nm_ values between the final and initial absorbance measurements was used for quantifying the intracellular L-Proline concentration. The L-Proline concentrations were normalized to the total protein concentration in each cell lysate sample, determined using a calibration plot of Bovine Serum Albumin (BSA) known protein concentration standards (0–2.0 mg mL^−1^) prepared in methanol. Protein standards or cell lysate protein samples (3 µL each) were mixed with 200 µL BCA Pierce Protein assay working solution in a 96-well microplate. Then, the microplate was covered in foil to protect samples from light and subjected to 37 °C static incubation for 30 min followed by a brief cooling-down period to room temperature. Lastly, the absorbance at 562 nm of the protein standards and samples was recorded in the plate reader (in sweep mode, endpoint absorbance reading).

### 2.18. Statistical Analysis

All data presented are representative of at least three repeated experiments and are presented as mean ± SEM unless described otherwise. The data were normally distributed, and comparisons between the groups were made using Student’s *t*-test wherever applicable. A *p*-value of <0.05 was considered statistically significant. All analyses were performed using the Prism 9.5.0 (GraphPad Inc., Boston, MA, USA) software unless mentioned otherwise.

## 3. Results

### 3.1. PYCR2 Expression Is Upregulated during Colon Carcinogenesis in Both Mice and Humans

**Figure 1:**
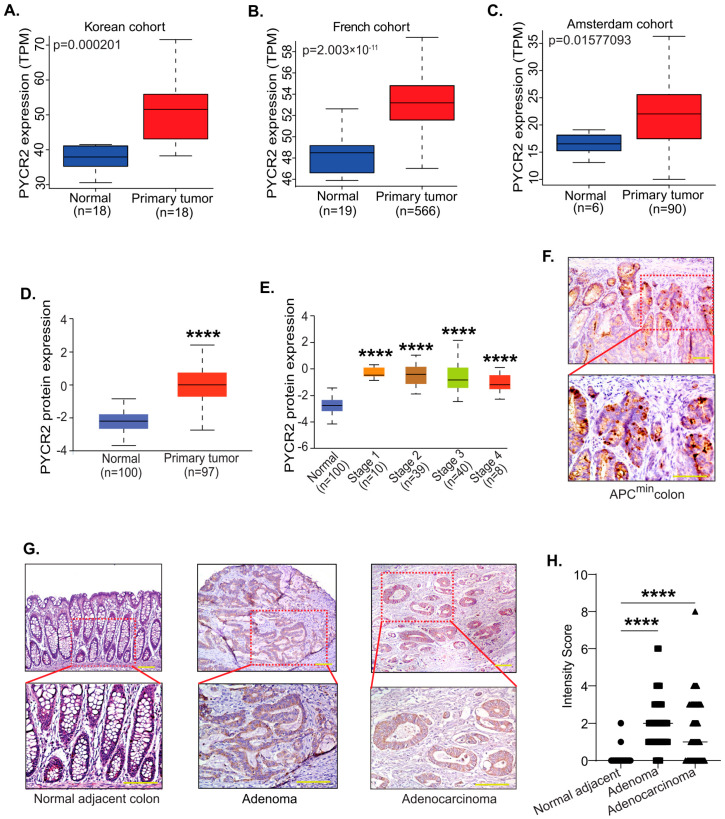
PYCR2 expression increases significantly in colon cancer. (**A**–**C**) PYCR2 mRNA expression in Asian and European CRC cohorts (*p* = 0.000201 for Korean cohort, *p* = 2.003 × 10^−11^ for French cohort, and *p* = 0.01577093 for Amsterdam cohort). (**D**) Analysis of PYCR2 protein expression in colorectal cancer patients in the CPTAC database (adjacent normal vs. primary tumor, *p* = 1.49 × 10^−43^). (**E**) PYCR2 protein expression in different stages of colorectal cancer (normal vs. stage 1, *p* = 6.5 × 10^−5^; normal vs. stage 2, *p* = 2.13 × 10^−22^; normal vs. stage 3, *p* = 2.94 × 10^−16^; and normal vs. stage 4, *p* = 5.49 × 10^−4^). (**F**) Representative image showing PYCR2 expression in colon tumors of APC^min^ mice. (**G**) Representative images of immunohistochemical analysis of PYCR2 expression in colon adenoma and adenocarcinoma in comparison to normal adjacent human colon. (**H**) Quantitative analysis of PYCR2 expression in human colon polyps and CRC samples. **** *p* < 0.0001.

To determine the status of PYCR mRNA expression in colon cancer, we first analyzed the Cancer Genome Atlas Program (TCGA) database using a publicly available software portal: UALCAN (The University of Alabama at Birmingham Cancer Data Analysis Portal). The mRNA expressions for all three PYCR isoforms (PYCR1, PYCR2, and PYCR3) were significantly upregulated in CRC compared with the adjacent normal colon, which was in accordance with recent reports [33]. We found a similar significant increase in PYCR2 expression in CRC patients in publicly available databases for Korean (*p* = 0.000201), French (*p* = 2.003 × 10^−11^), and Amsterdam (Amsterdam, *p* = 0.015) cancer patients [22] (Figure 1A–C). Further analysis of the protein expression using CPTAC (Clinical Proteomics Tumor Analysis Consortium) showed a similar significant upregulation of PYCR2 expression in CRC (Figure 1D,E). Together, these data supported a universal increase in PYCR2 expression in CRC. Interestingly, in a further analysis of the association with patient survival, only PYCR2 expression—among PYCR1, PYCR2, and PYCR3—showed a significant association with poor patient survival (Figure S1A–C).

To validate the data obtained from the in-silico analysis of the public databases, we examined PYCR2 expression in the colon of APC^min^ (Adenomatous Polyposis Coli) mice, the widely used mouse model of CRC [34,35]. Additionally, human colon polyps and adenocarcinoma specimens were utilized. Immunohistochemical (IHC) analysis was performed using an anti-PYCR2 antibody. Microscopical examination revealed a robust increase in PYCR2 expression in the tumors compared with the adjacent normal mice colon tissue, suggesting that an increase in PYCR2 is an early event in CRC (Figure 1F). A similar trend was also observed in human colon adenomas (*p* < 0.0001) and adenocarcinomas (*p* < 0.0001), supporting the role of PYCR2 in CRC development (Figure 1G,H).

### 3.2. PYCR2 Upregulation in CRC Is an Early and Universal Event across CRC Phenotypes

**Figure 2:**
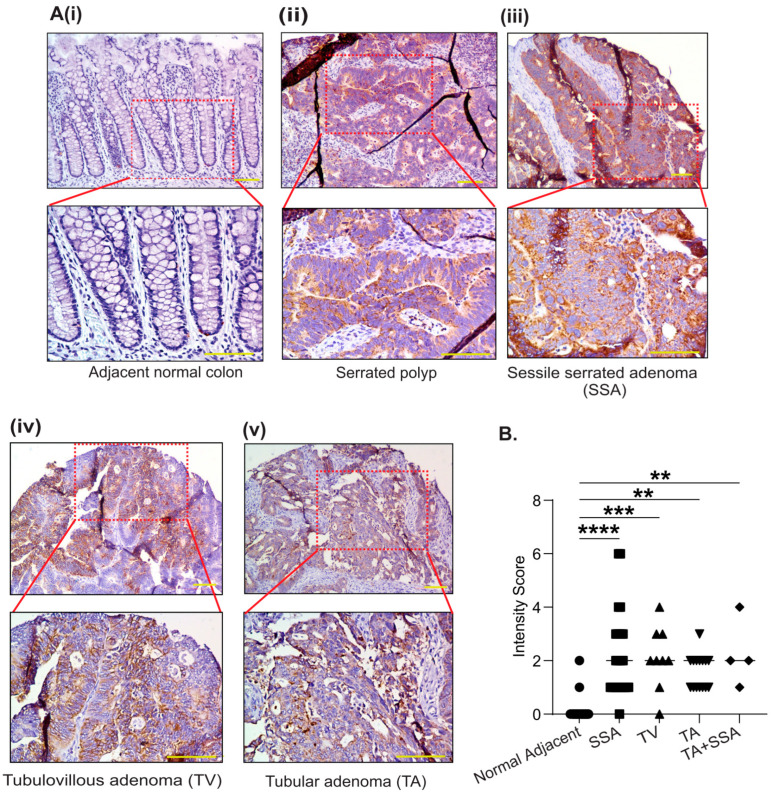
PYCR2 protein expression is significantly upregulated in all histological types of CRC adenomas. (**Ai**–**Av**) Representative images of immunohistochemical analysis of PYCR2 expression in different types of colon adenomas and adjacent normal colon (TMA, N#109). (**B**) Scoring analysis of PYCR2 immunostaining intensity in normal adjacent colon vs. colon adenoma (*p <* 0.0001 for SSA, *p* = 0.002 for TV, *p* = 0.0015 for TA, and TA + SSA respectively). The data are presented as mean + SEM. Statistical significance was determined using one-way ANOVA and a post hoc Tukey’s test for pairwise comparison. **** *p* < 0.0001, *** *p* < 0.001, and ** *p* < 0.01.

Colon adenomas can have different growth patterns and thus differing disease aggressiveness and prognoses [36]. Therefore, to examine if PYCR2 expression in CRC is associated with a specific adenoma type, we utilized adenomas and adjacent normal colons from CRC patients to determine if increased PYCR2 expression is associated with a specific CRC type. We included diverse histological types of colon adenomas including tubulovillous (TVA), serrated (SA), sessile serrated (SSA), and tubular adenoma (TA), as these histological subtypes are associated with variable aggressiveness in colon cancer [37]. A blinded analysis of the PYCR2 staining intensity and subcellular localization was performed by a gastrointestinal pathologist. As shown in Figure 2(Ai–Av), an elevated PYCR2 expression was observed in the colon adenomas, which was significant compared with PYCR2 expression in adjacent normal colons. However, we found no major differences in PYCR2 expression between different histological subtypes of colon adenomas by quantifying the PYCR2 staining intensity score (Figure 2B). Overall, these data support the outcome of the in-silico analysis, which suggests a positive association between PYCR2 expression and colon carcinogenesis.

### 3.3. Genetic Manipulation of PYCR2 Expression in Colon Cancer Cells Modulates Epithelial-to-Mesenchymal Transition (EMT) and Their Tumorigenic and Invasive Abilities

**Figure 3:**
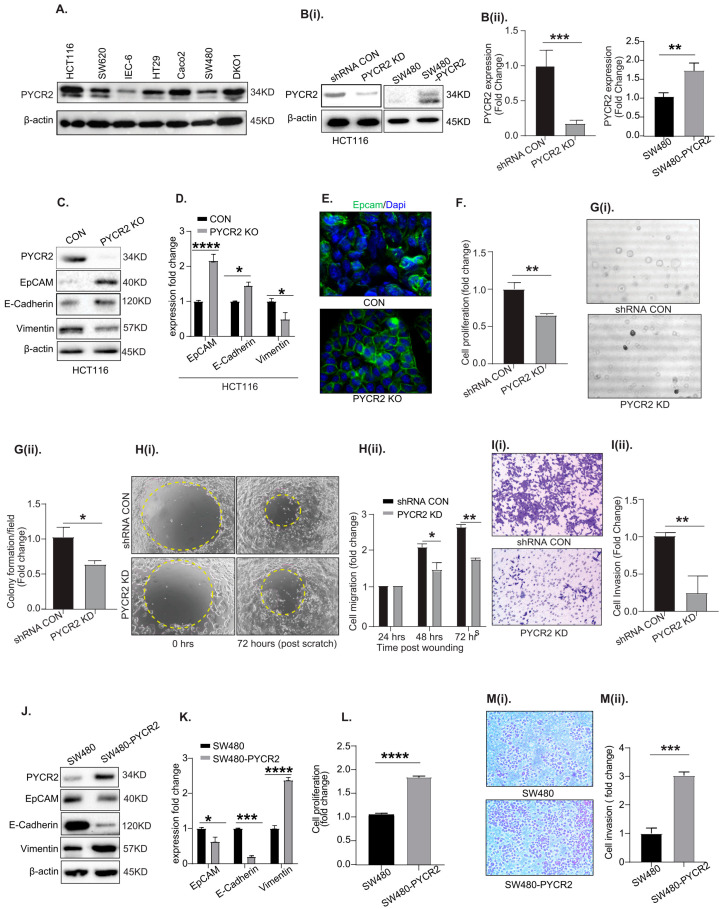
Genetic manipulation of PYCR2 expression modulates oncogenic properties of CRC cells. (**A**) Western blot analysis of PYCR2 expression in different CRC cell lines. The IEC-6 cells served as normal intestinal epithelial cells. (**Bi**,**Bii**) Immunoblot analysis of control and genetically manipulated PYCR2 HCT116 and SW480 cells and densitometric analysis (*p* = 0.0021 and *p* = 0.015). (**C**,**D**) Representative immunoblot analysis and densitometric analysis of EpCAM, E-cadherin, and vimentin in control and PYCR2-KO HCT116 cells (*p* = 0.00012 for EpCAM and *p* = 0.021 and 0.019 for E-cadherin and vimentin). (**E**) Immunofluorescence staining images for EpCAM expression in control and PYCR2-KO HCT116 cells. (**F**) Cell proliferation assays using the HCT116 control and PYCR2-KD cells (*p <* 0.0001), (**Gi**,**Gii**) Soft agar assay using the HCT116 control and PYCR2-KD cells (*p* = 0.0257), (**Hi**,**Hii**) Cell migration assay using the HCT116 control and PYCR2-KD cells (*p* = 0.0355 at 48 h and *p* = 0.0048 at 72 h), (**Ii**,**Iii**) Cell invasion in HCT116 control and PYCR2-KD cells (*p* = 0.0017), and quantitative analysis. (**J**,**K**) Representative images of the immunoblot analysis of EpCAM, E-cadherin, and vimentin in control and PYCR2-overexpressing SW480 cells and densitometric evaluation (*p* = 0.031 for EpCAM and *p* = 0.0015 and 0.00029 for E-cadherin and vimentin). (**L**) Representative data for the effect of PYCR2 overexpression on cell proliferation (*p* = 0.00019). (**Mi**,**Mii**) Representative data for the cell invasion (*p* = 0.0024) in control and PYCR2-overexpressing SW480 cells. Data are presented as mean + SEM. Statistical significance was determined using Student’s *t*-test and one-way ANOVA. * *p* < 0.05, ** *p* < 0.01, *** *p* < 0.001, and **** *p* < 0.0001.

To further investigate the causal role of PYCR2 in colon carcinogenesis, we genetically manipulated endogenous PYCR2 expression. In this regard, we first examined PYCR2 expression in a panel of colon cancer cell lines. IEC-6 (intestinal epithelial cells) cells served as a normal intestinal epithelial cell line [23]. As shown in Figure 3A, the immunoblot analysis showed robust PYCR2 expression in all the CRC cell lines compared with the IEC-6 cells. HCT116 and SW620 cells were selected for further investigation based on high endogenous PYCR2 expression and their known tumorigenic and metastatic properties [38]. SW480 and HT29 cells were selected for PYCR2 overexpression given their relatively low endogenous PYCR2 expression.

**Figure 4:**
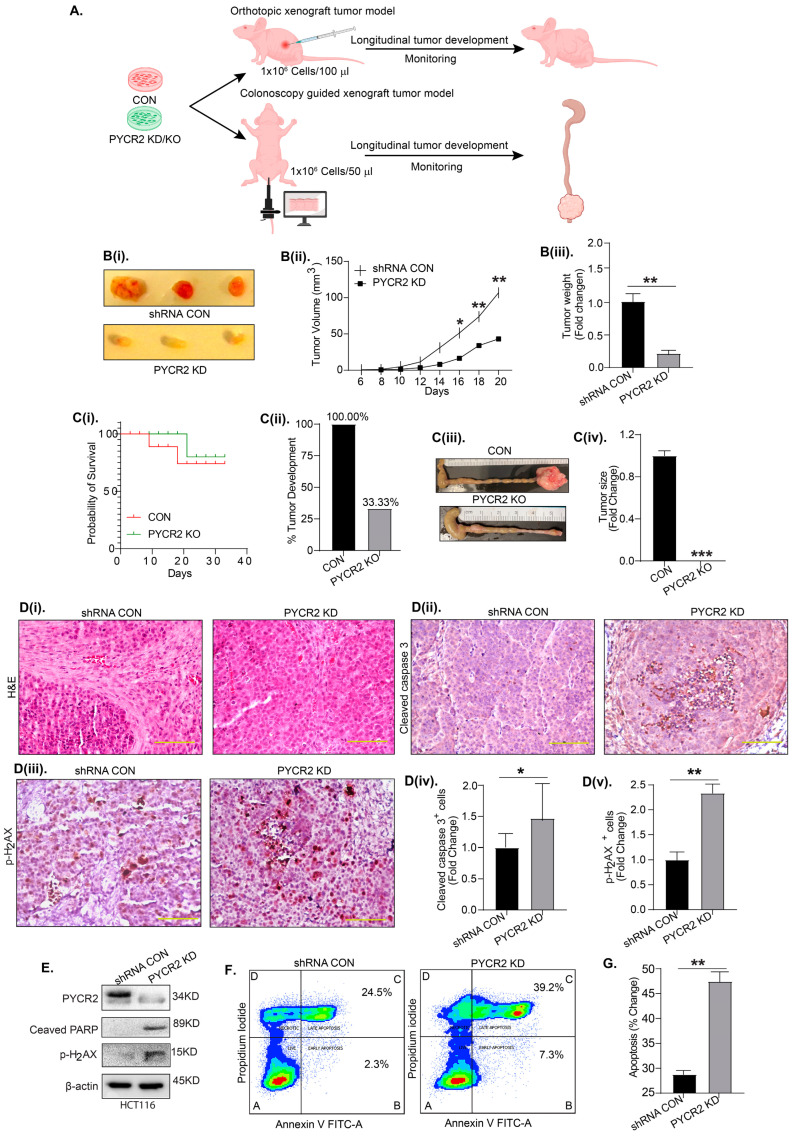
Inhibition of PYCR2 expression inhibits xenograft tumor growth and promotes apoptosis. (**A**) Schematics of in vivo studies using murine models of subcutaneous xenograft tumor growth and colonoscopy-guided cancer cell transplantation into the colon wall. (**Bi**) Representative images of the tumors isolated from athymic/nude mice injected with control or PYCR2-inhibited SW620 cells. (**Bii**,**Biii**) Statistical analysis showing % change in tumor volume (*p* = 0.0048) and fold change in tumor weight (*p* = 0.0019). (**Ci**) The analysis of the probability of mouse survival after colonoscopy-guided injection. (**Cii**–**Civ**) Representative images of the quantification of the % of tumor development; respective images of colon tumors and tumor size quantification (*p* = 0.0014, control vs. PYCR2 KD). (**Di**) Representative H&E images of the tumors. (**Dii**–**Dv**) Representative images of IHC using anti-cleaved caspase-3 and p-H_2_AX antibodies in xenograft tumors and quantitative analysis (*p* = 0.0349 and *p* = 0.0018). (**E**) Immunoblot analysis for p-H_2_AX and cleaved PARP in HCT116 control and PYCR2-KD cells. (**F**,**G**) FACS analysis for early and late apoptosis in HCT116 control and PYCR2-KD cells and quantitative analysis (*p* = 0.0085). The representative figure has four quadrants where A = live cells, B = early apoptosis, C = late apoptosis, and D = necrosis. Data are presented as mean + SEM, and significance was determined using Student’s *t*-test and one-way ANOVA. * *p* < 0.05, ** *p* < 0.01, and *** *p* < 0.001.

To inhibit PYCR2 expression, we utilized siRNA (Figures S2A,D and S4A); a shRNA plasmid construct (Figure 3(Bi), Figure 4E, Figure S5A and Figure 6B,E); and CRISPR-cas9 (Figure 3C, Figure 7(Ci) and Figure 8(Ai)). We also utilized inducible the (Doxycycline-mediated)-shRNA-mediated silencing of PYCR2 expression (Figure S2J). For PYCR2 overexpression in SW480 and HT29 cells, we used an expression plasmid construct, pcDNA3-PYCR2, with a CMV promoter. Immunoblotting, followed by densitometric analysis, was performed to validate the loss or overexpression of PYCR2 expression in HCT116 and SW480 cells (Figure 3(Bi,Bii)). RT-qPCR was performed to confirm the inhibition of *PYCR2* mRNA expression caused by different genetic tools (Figure S2B,E,H,K).

Effects on EMT (epithelial to mesenchymal transition), cell proliferation, anchorage-independent growth, cell migration, and cell invasion were determined. An immunoblot analysis of EpCAM, E-cadherin (known epithelial cell markers), and vimentin (known mesenchymal cell markers) was performed to determine the effect of PYCR2 upon cancer cell phenotype [39,40,41]. Loss of PYCR2 resulted in the significant upregulation of EpCAM and E-cadherin expressions, while vimentin expression was downregulated, suggesting mesenchymal-to-epithelial transition (MET) (Figure 3C,D). An immunofluorescence analysis further showed the membrane localization of EpCAM in PYCR2-KO HCT116 cells (Figure 3E). The inhibition of PYCR2 expression in SW620 cells resulted in similar outcomes (Figure S5C). The genetic inhibition of PYCR2 expression also resulted in the significant inhibition of cell proliferation (Figure 3F and Figure S2C,F,I,L), growth in soft agar (Figure 3(Gi,Gii)), and migration (Figure 3(Hi,Hii)). Cell invasion was also significantly inhibited in PYCR2-inhibited cells (Figure 3(Ii,Iii)).

The complementary studies of SW480 and HT29 cells overexpressing PYCR2 showed contrasting inhibition of EpCAM and E-cadherin expressions, while vimentin expression was upregulated (Figure 3J,K and Figure S5E). Additionally, PYCR2-overexpressing cells showed significant increases in cell proliferation (Figure 3L and Figure S5F), cell invasion (Figure 3(Mi,Mii)), and cell migration abilities (Figure S5(Gi,Gii)). Overall, the above data validated the critical role of PYCR2 expression in promoting EMT and the tumorigenic abilities of CRC cells.

### 3.4. Loss of PYCR2 Expression Decreases Intracellular Proline Content in CRC Cells

PYCR2 is a key enzyme of the proline biosynthetic pathway; thus, we examined whether the loss of PYCR2 was sufficient to decrease proline levels in colon cancer cells. HCT116 control cells and PYCR2-KO cells were cultured in a complete culture medium with proline (RPMI) and without proline (DMEM). As shown in Figure S3A–F, intracellular proline levels in HCT116 control cells (CRISPR CON) were ~110 µM in DMEM and nearly 400 µM in RPMI. The PYCR2-KO cells showed a significant decrease in proline content relative to the control cells in both types of culture media. Overall, these data support the critical role of PYCR2 in regulating intracellular proline levels in CRC despite PYCR1 and PYCR3 also potentially contributing to proline biosynthesis.

### 3.5. PYCR2 Inhibition Significantly Inhibits In Vivo Tumor Growth

Given that PYCR2 inhibition resulted in the significant inhibition of the proliferation and invasive motility of CRC cells, we next determined the effects of PYCR2 loss on in vivo tumor growth. The subcutaneous injection and intramucosal transplantation of control and PYCR2-inhibited colon cancer cells were utilized for the in vivo tumor growth studies (Figure 4A). For the subcutaneous xenograft tumor growth assay, athymic/nude mice (6–8 weeks old) were injected with a single-cell suspension (1 × 10^6^/100 μL) of control and PYCR2-KD SW620 cells under the dorsal flank. As shown in Figure 4Bi, tumor size in mice receiving the PYCR2-KD cells was considerably lower compared with mice receiving the control cells. At the study termination, tumors in mice injected with PYCR2-KD cells were significantly smaller in size (*p* = 0.0048) and weight (*p* = 0.0019) relative to the tumors from mice injected with the control cells (Figure 4(Bii,Biii)).

To further determine the role of PYCR2 in colon carcinogenesis in a true colonic microenvironment, we used an orthotopic xenograft model recently described by our lab, where cancer cells are transplanted into the colonic wall to generate tumors [29]. A colonoscopy-guided intramucosal cell transplantation was performed on athymic/nude mice (8 weeks old). As shown in Figure 4(Ci), the mice injected with PYCR2-KO HCT116 cells showed a higher survival probability compared with mice receiving the control HCT116 cells. When sacrificed, mice that received the HCT116 control cells showed remarkably large tumors in their colons compared with mice injected with PYCR2-KO cells. The tumors in mice transplanted with PYCR2-KO cells were very small or almost negligible (Figure 4(Cii–Civ). Taken together, these data supported the role of PYCR2 in promoting colon cancer.

A histological evaluation of the H&E slides showed a compact and cuboidal-epithelial-cell-like morphology in the tumors generated by the PYCR2-KD cells compared with the diffused and irregular cell architecture in tumors produced by control SW620 cells (Figure 4(Di)). Subsequent IHC staining of the tumor tissue sections further showed a significantly high number of cleaved caspase-3 and p-H_2_AX (a DNA damage marker)-positive cells in tumors from PYCR2-KD cells (Figure 4(Dii–Dv)). For additional validation showing that an increase in cancer cell apoptosis in response to PYCR2 inhibition may be responsible for the inhibition of tumor growth, we performed immunoblot analysis using lysates from HCT116 and SW620 control cells and PYCR2-KD cells. As shown in Figure 4E and Figure S4B, PYCR2-KD in both cells promoted the expression of cleaved PARP and p-H2AX compared with the control cells. A similar increase in apoptosis markers was observed in these cells upon siRNA-mediated PYCR2 silencing in HCT116 cells (Figure S4A). FACS-based analysis further showed a significant increase in both early and late apoptosis in PYCR2-KD cells compared with control cells (*p* = 0.0085) (Figure 4F,G). Overall, the above data supported the causal role of PYCR2 in promoting colon tumorigenesis by promoting cancer cell survival and proliferation.

### 3.6. LC-MS/MS-Based Proteomics Analysis Showed the Profound Effects of PYCR2 Loss in Cell Survival and Proliferative and Metabolic Pathways

**Figure 5:**
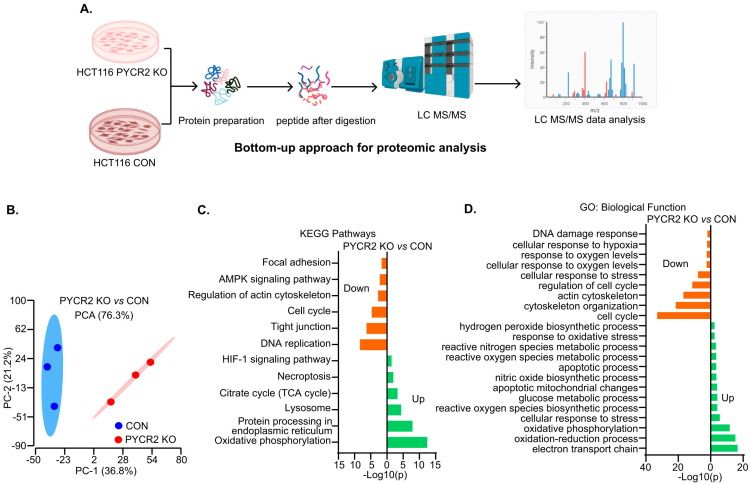
LC-MS/MS proteomics analysis to determine effects of PYCR2 loss of expression. (**A**) Schematics of the LC-MS/MS proteomics analysis. (**B**) Principal component analysis of the proteins differentially expressed in the control and PYCR2-KO HCT116 cells. (**C**,**D**) Analyses of the KEGG pathway and the GO biological function for differentially expressed proteins in PYCR2-KO versus control cells.

In further studies, to determine how PYCR2 promotes CRC, we performed an unbiased LC-MS/MS proteomics analysis. HCT116 control cells and PYCR2-KO cells were used (Figure 5A). The unsupervised hierarchical clustering of the outcome confirmed that more than 5000 proteins were differentially expressed between the control cells and PYCR2-KO cells. A principal component analysis (PCA) further separated the control and KO cells as distinct entities, demonstrating the profound effect of PYCR2 on cellular homeostasis (Figure 5B).

To understand the functional profiles of proteins differentially expressed by the loss of PYCR2, we further performed KEGG pathway and GO biological function analyses. As shown in Figure 5C, PYCR2-KO cells showed downregulation in AMPK signaling, the cell cycle, and DNA replication, which play essential roles in oncogenesis. Control cells exhibited an enhanced TCA cycle and oxidative phosphorylation, which significantly contribute to CRC tumorigenesis (Figure 5D). The outcomes obtained from the GO biological function analysis largely aligned with the KEGG pathway analysis (Figure 5D). Overall, these data suggested the critical role of PYCR2 in cellular metabolism and proliferative mechanisms.

### 3.7. Loss of PYCR2 Reduces Cell Proliferation and Inhibits Cancer Stem Cell Populations

**Figure 6:**
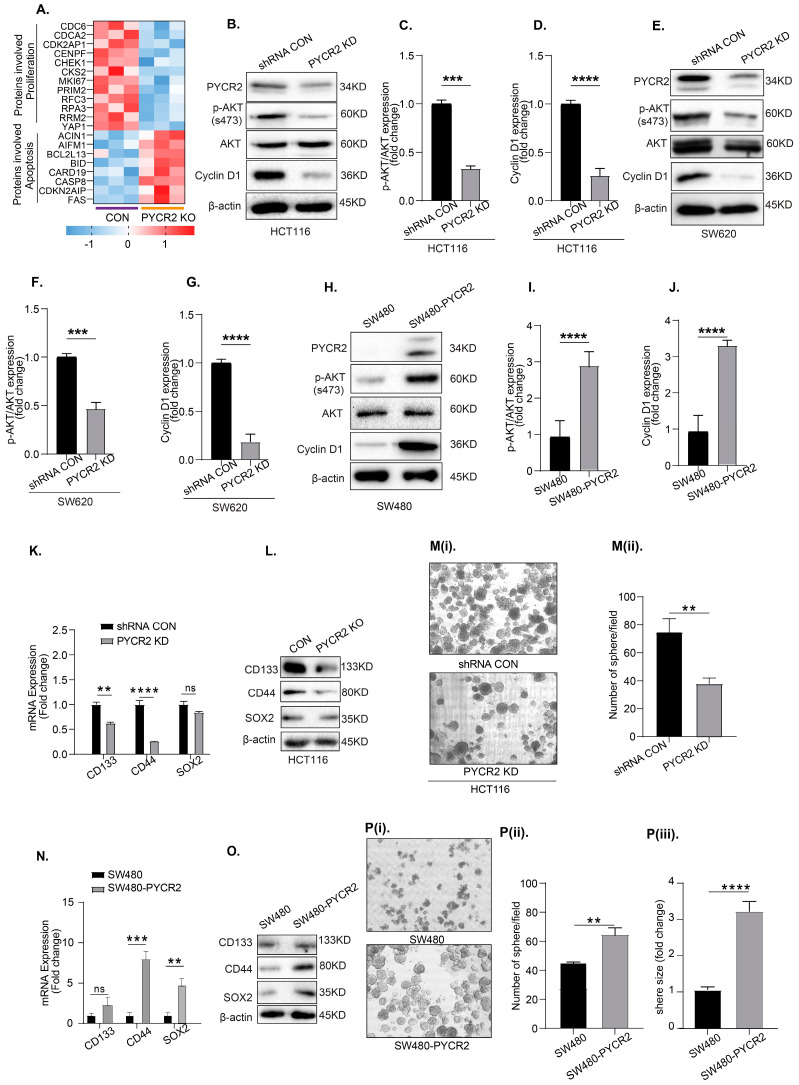
PYCR2 regulates cell survival pathways and cancer stem cell population. (**A**) Heatmap analysis of proteins involved in cell apoptosis and proliferation. (**B**–**G**) Immunoblotting and densitometric analysis examining the expression of p-AKT and cyclin D1 in control and PYCR2-inhibited HCT116 and SW620 cells. (**H**–**J**) Immunoblotting and densitometric analysis examining the expression of p-AKT and cyclin D1 in control and PYCR2-overexpressing SW480 cells. (**K**) mRNA expression analysis for colonic CSC markers in HCT116 control cells and PYCR2-KD cells (*p <* 0.011 for CD133, and *p* = 0.00014 for CD44 and 0.9484 for Sox2 (ns). (**L**) Representative immunoblots for the analysis of colonic CSC markers in control and PYCR2-KO HCT116 cells. (**Mi**,**Mii**) Sphere-forming assay using HCT116 control cells and PYCR2-KD cells and quantitative analysis (*p* = 0.0018). (**N**) mRNA expression analysis of colonic CSC markers in control and PYCR2-overexpressing SW480 cells (*p* < 0.769 for CD133 (ns), 0.00156 for CD44, and *p <* 0.0001 for Sox2). (**O**) Representative immunoblots for the analysis of colonic CSC markers in control and SW480-PYCR2 cells. (**Pi**–**Piii**) Sphere-forming assay using control and SW480-PYCR2 cells and quantitative analysis (*p* = 0.0158 for number of spheres, and *p* = 0.00013 for size). Data are presented as mean + SEM. Statistical significance was determined using Student’s *t*-test and one-way ANOVA. ns = non-significant, ** *p* < 0.01, *** *p* < 0.001, and **** *p* < 0.0001.

A subsequent heatmap analysis of LC-MS/MS proteomics data showed that proteins associated with apoptosis were upregulated, while proteins associated with cell proliferation were significantly downregulated (Figure 6A). To validate these findings, we performed immunoblotting using lysates from the control and PYCD2-KD CRC cells.

Here, we first determined possible changes in the markers of cell proliferation/survival pathways, as these pathways were affected by the dysregulation of proline metabolism [42,43]. We used the expression of cyclin D1 and p-AKT (s473), as these pathways are upregulated in cancers and promote oncogenic growth [44]. Remarkably, both cyclin D1 and p-AKT expressions were downregulated in PYCR2-KD cells compared with the control cells (Figure 6B–G). In contrast, SW480-PYCR2 cells showed upregulation in both p-AKT(s473) and cyclin D1 expression (Figure 6H–J).

Cancer progression, including CRC, has been linked with the enrichment of cancer stem cells (CSCs) [45,46,47]. We thus further analyzed if the expression of colon cancer stem cell markers is also differentially expressed in PYCR2-manipulated CRC cells. An RT-qPCR was performed using gene-specific primers for CRC-associated CSC biomarkers *CD133*, *CD44*, and *SOX2* [48,49,50]. As shown in Figure 6K, the expression of all these markers was significantly downregulated in PYCR2-inhibited cells. Immunoblot analysis further confirmed similar downregulation in CD133, CD44, and SOX2 proteins in HCT116-KO cells (Figure 6L). Immunoblotting using control and PYCR2-inhibited SW620 cells showed similar downregulation in cancer stem cell markers (Figure S5C). The sphere-formation assay, an established functional model of CSCs, [24,51] also showed significant downregulation in PYCR2-KD cells compared with the control cells (Figure 6(Mi,Mii)).

Complementary studies using SW480-PYCR2 cells showed contrasting upregulation in mRNA expressions for *CD133*, *CD44*, and *SOX2* (Figure 6N). An immunoblot analysis further showed an increase in the expression of these proteins (Figure 6O). We also found significant increases in the number and the size of the sphere in SW480-PYCR2 cells compared with SW480-control cells (Figure 6(Pi–Piii)). Overall, the above data suggested that PYCR2 promotes CSCs in promoting CRC.

### 3.8. Loss of PYCR2 Inhibits MASTL/Wnt Signaling in Its Tumorigenic Promoting Effects

**Figure 7:**
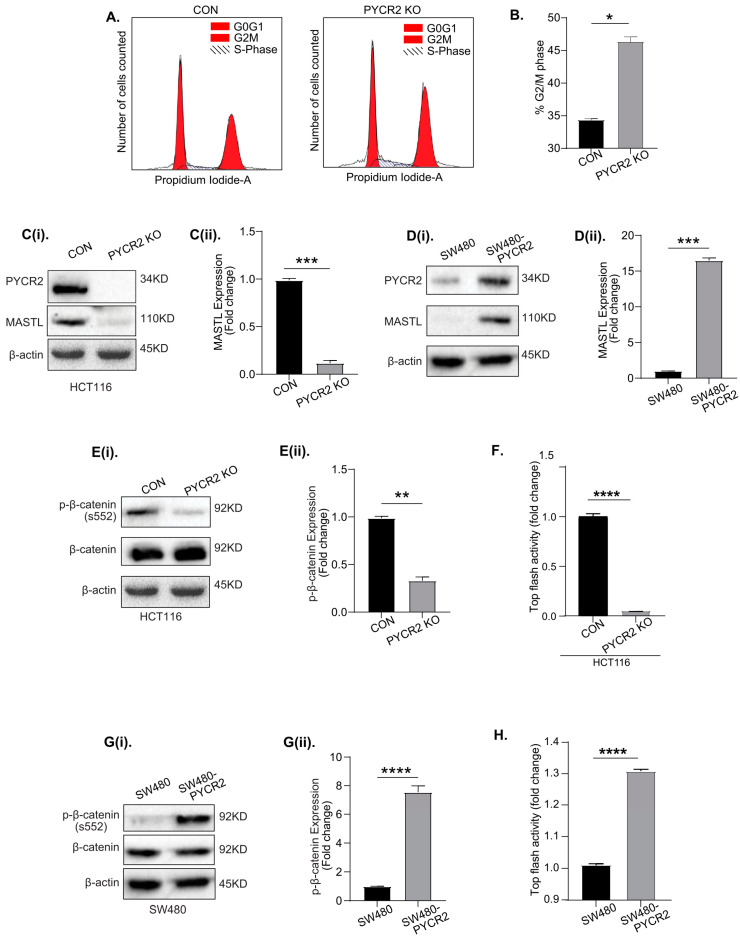
PYCR2 regulates the cell cycle and modulates MASTL/Wnt/β-catenin signaling. (**A**,**B**) Representative images of the cell cycle analysis using the control and PYCR2-KO HCT116 cells showing cell cycle arrest in PYCR2-KO cells at the G2/M phase and the subsequent quantification of the % of cells arrested at the G2/M phase. (**Ci**,**Cii**) Representative images of immunoblots and densitometric analysis examining the effects of PYCR2 on MASTL expression in PYCR2-KO HCT116. (**Di**,**Dii**) Immunoblots and densitometric analysis for MASTL expression in control and SW480-PYCR2 cells. (**Ei**,**Eii**) Representative images of immunoblots and densitometric analysis examining the effects of PYCR2 on Wnt signaling (p-β catenin s552) using the control and PYCR2-KO HCT116. (**F**) TOP-flash luciferase-based analysis of control and PYCR2-KO HCT116 cells. (**Gi**,**Gii**) Effect of PYCR2 overexpression on Wnt signaling (p-β catenin s552) in control and SW480-PYCR2 cells followed by densitometric analysis. (**H**) TOP-flash activity analysis of control and SW480-PYCR2 cells. Data are presented as mean + SEM. Statistical significance was determined using Student’s *t*-test and one-way ANOVA. * p < 0.05, ** p < 0.01, *** *p* < 0.001, and **** *p* < 0.0001.

Our proteomics data suggested the potential role of PYCR2 in regulating the cell cycle and CRC cell proliferation (Figure 5C,D). To inquire further into this, we performed a cell cycle analysis using flow cytometry. As shown in Figure 7A,B, the loss of PYCR2 indeed arrested the cell cycle at the G2/M phase. Of note, the inhibition of MASTL, highly upregulated in CRC, results in similar inhibition in the cell cycle at the G2/M phase [27]. We further demonstrated that MASTL overexpression promotes colon cancer aggressiveness by promoting cancer stem cells, similar to PYCR2 [27]. Thus, we determined if PYCR2 regulates MASTL expression to promote CRC. An immunoblot analysis was performed using total cell lysates, which, indeed, showed the sharp downregulation of MASTL expression in PYCR2-inhibited colon cancer cells (Figure 7(Ci,Cii) and Figure S5A). To confirm this interesting outcome, we examined if the overexpression of PYCR2 would promote MASTL expression. Indeed, as shown in Figure 7(Di,Dii) and Figure S5D, PYCR2 overexpression promoted MASTL expression in SW480 and HT29 cells. Overall, the above data suggested that PYCR2 regulates MASTL expression, which is known to promote CRC.

The Wnt/β-catenin signaling pathway is implicated in promoting CRC oncogenesis [52]. We previously reported that MASTL modulates Wnt/β-catenin signaling to promote CRC [27]. Thus, to determine if the modulation of PYCR2 expression also affects Wnt/β-catenin signaling, we examined the expression of p-β-catenin(s552), which is associated with the activation of Wnt signaling [53]. As shown in Figure 7(Ei,Eii) and Figure S5A, an immunoblot analysis showed that the expression of p-β-catenin(s552) was sharply downregulated in response to the loss of PYCR2 expression. Luciferase-based Wnt reporter activity (TOP-flash assay) further showed that luciferase activity was significantly reduced in PYCR2-KO HCT116 cells compared with the control cells (Figure 7F). In contrast, the expression of p-β-catenin(s552) was significantly upregulated in SW480-PYCR2 and HT29-PYCR2 (PYCR2 overexpression) cells (versus control cells; Figure 7(Gi,Gii) and Figure S5D). PYCR2 overexpression also promoted TOP-flash activity (Figure 7H). Overall, these data suggested a causal link between PYCR2-MASTL and Wnt signaling in promoting CRC.

### 3.9. MASTL Mediates the CRC-Promoting Effects of PYCR2 Expression

**Figure 8:**
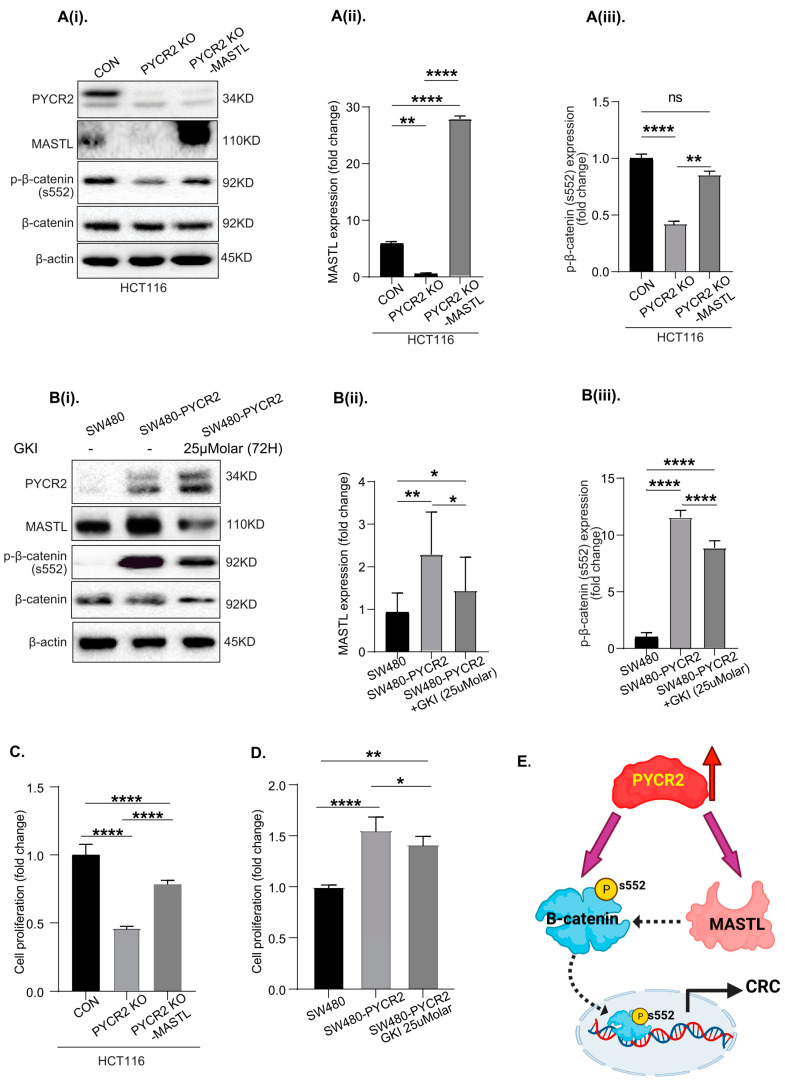
MASTL mediates CRC-promoting effects of PYCR2 expression. (**Ai**–**Aiii**) Immunoblot analysis determining the effects of MASTL overexpression in PYCR2-KO HCT116 cells and densitometric analysis of MASTL and p-βcatenin s552 expression in control, PYCR2 KO, and MASTL overexpression in PYCR2 KO HCT116 cells. (**Bi**–**Biii**) Immunoblot analysis determining the effect of the GKI-an inhibitor on MASTL expression/activity in PYCR2-overexpressing SW480 cells. A densitometric analysis of MASTL and p-βcatenin s552 expression in control, PYCR2 overexpression, and MASTL-inhibited SW480 cells is also presented. (**C**,**D**) Cell proliferation assay of HCT116-KD and SW480-PYCR2 cells after MASTL overexpression and inhibition, respectively. (**E**) Schematics summarizing our findings on the regulatory role of PYCR2 in CRC progression caused by modulating MASTL/Wnt/β-catenin signaling. Data are presented as mean + SEM. Statistical significance was determined using Student’s *t*-test and one-way ANOVA. ns = non-significant, * *p <* 0.05, ** *p* < 0.01 and **** *p* < 0.0001.

We previously reported that MASTL regulates CRC progression by regulating cancer cell apoptosis in a manner dependent on Wnt/β-catenin signaling [27]. Thus, in light of the above data, we further examined if overexpressing the MASTL protein in PYCR2-inhibited CRC cells would reduce the effects of PYCR2 loss. Transient transfection was performed, and the effect on MASTL, PYCR2, and p-β-catenin (s552) expression was determined. As shown in Figure 8(Ai–Aiii), the overexpression of MASTL did not affect PYCR2 expression; however, it promoted Wnt/β-catenin signaling. Similar results were found in SW620 PYCR2 KD cells in response to MASTL overexpression (Figure S5B). To further validate this finding, in complementary studies, we inhibited MASTL expression/activation in SW480-PYCR2 cells by treating the cells with a MASTL inhibitor (GKI). As shown in Figure 8(Bi–Biii), Wnt/β-catenin signaling was inhibited in GKI-treated, SW480-PYCR2-overexpressing cells compared with untreated PYCR2-overexpressing cells. Further analysis also showed that manipulating MASTL expression also reverted the effects of PYCR2 expression in respective CRC cell lines. Complementary studies were conducted to determine cell proliferation with MASTL overexpression or inhibition in PYCR2-manipulated cells. As shown in Figure 8C, PYCR2 KO significantly reduced cell proliferation; however, the rescue effect was observed after MASTL overexpression in PYCR2 KO HCT116 cells. Similarly, the MASTL inhibitor (GKI) treatment in SW480-PYCR2 cells did not show detrimental effects on cell proliferation in comparison with untreated SW480-PYCR2 cells (Figure 8D). Taken together, the above data supported the causal role of PYCR2 in modulating MASTL/Wnt/β-catenin signaling in regulating colon carcinogenesis. The schematics in Figure 8E summarize our findings on the regulatory role of PYCR2 in CRC progression caused by modulating MASTL/Wnt/β-catenin signaling.

## 4. Discussion

The rewiring of normal cellular metabolism by cancer cells for their survival and, thus, cancer progression has been widely documented [54]. In this regard, the causal role of proline metabolism in promoting cancer cell survival and invasive mobility has been demonstrated [5]. However, its therapeutic targeting has been challenging due primarily to the complexity of its regulation. In our current study, we demonstrate, based on comprehensive in silico and biochemical in vitro and in vivo studies, the role of PYCR2 in regulating colon carcinogenesis. Our results are strongly supported by recent reports showing an association between PYCR2 expression and CRC aggressiveness and poor prognoses [33,55]. However, our study builds on these initial findings and not only confirms the causal role of PYCR2 in promoting tumorigenicity and the invasive mobility of colon cancer cells in vitro but also in vivo oncogenic growth by regulating MASTL/Wnt/β-catenin signaling. Overall, the outcome of the current study identifies PYCR2 as a novel biomarker of colon cancer progression and poor prognosis and a potential therapeutic target.

Notably, our initial analysis suggested the important role of PYCR2 in promoting CRC aggressiveness, as only PYCR2, out of PYCR1, PYCR2, and PYCR3/PYCRL, showed a significant association with poor patient survival. A similar outcome was reported by recent studies [33,55] Our data from a protein expression analysis of the CPTAC database and an IHC analysis of CRC adenomas and adenocarcinomas supported a significant increase in PYCR2 levels in colon cancer but also demonstrated that it is an early event. Our finding that PYCR2 expression was not significantly different between histological CRC subtypes further implies that an increase in PYCR2 is a common event in CRC. The data from the Asian and European cancer cohorts further support such an assumption. Overall, our data support findings from recent studies suggesting an association between PYCR2 and colon cancer aggressiveness [33,55].

Our additional analysis using the genetic “loss-of-PYCR2-expression” approach further revealed that an increase in PYCR2 expression in CRC is causally related to promoting colon carcinogenesis. In this regard, we obtained reproducible outcomes from two different CRC cell lines, HCT116 and SW620, finding that inhibiting PYCR2 inhibits cell survival, anchorage-independent growth, and invasive mobility, as well as in vivo tumor growth. Notably, both cell lines are highly tumorigenic and metastatic in nature [56,57] and express higher amounts of PYCR2 compared with normal colon cells. The rigor of these data is strong, as we inhibited PYCR2 expression using transient and stable genetic inhibition tools, including inducible inhibition and CRISPR-Cas9-mediated gene knockout approaches. The outcome of SW480 and HT29 cells overexpressing PYCR2 complemented the findings on the PYCR2-inhibited CRC cells. An unbiased LC-MS/MS proteomics analysis further demonstrated the critical role of PYCR2 in maintaining CRC homeostasis, as PYCR2 loss significantly inhibited metabolic, proliferative, and DNA-replicative pathways. Overall, our data supported the causal role of PYCR2 in promoting CRC by regulating cellular metabolism and oncogenic properties.

Our mechanistic findings on changes in molecular and signaling pathways, such as how PYCR2 loss may affect CRC cell biology, showed marked downregulation in the cellular contents of proline. Moreover, our data showed that PYCR2 regulates p-Akt expression in CRC cells, supported by a recent study that found that PYCR2 activates PI3K/AKT signaling in colon cancer cells [33]. Our data further showed that the loss of PYCR2 expression induces apoptosis in CRC cells, as the expression of established markers of cell apoptosis, cleaved PARP and pH_2_AX, were upregulated in PYCR2-inhibited cells [58]. A similar increase in these proteins in xenograft tumors further supported the role of PYCR2 in promoting cancer cell survival to then promote CRC. Our data, using FACS-based analysis, strengthened this assumption. An inverse association between p-Akt expression and apoptosis in PYCR2-manipulated cells further supported a positive correlation between PYCR2 expression and CRC cell survival. Overall, these data suggested that inhibiting PYCR2 expression inhibits CRC by dysregulating cell survival pathways and promoting cell death.

CSCs play a critical role in cancer progression, including CRC [45]. Our mRNA expression analysis data showed that the expression of *CD133*, *CD44*, and *SOX2* established CSC markers in CRC, which were altered because of PYCR2 loss, thus supporting the assumption that PYCR2 expression may cause the CSC niche to promote CRC aggressiveness. Our data showed that PYCR2 expression is associated with poor prognosis in CRC, and its inhibition promotes cancer cell death; oncogenic growth supports such a hypothesis. Our findings show that PYCR2 inhibition inhibits the MASTL/Wnt/β-catenin signaling cascade, which is also in sync with the possibility that PYCR2 expression promotes CRC by promoting CSC niches. In this regard, the role of Wnt signaling in promoting CRC is well documented, including promoting the CSC phenotype [59]. The role of MASTL in promoting cancer aggressiveness and therapy resistance is also well documented, including in CRC, in which we previously demonstrated that MASTL regulates CRC cell survival and CSC niches in a manner dependent on Wnt signaling [27]. The key phenotype obtained in this study was the regulation of cancer cell apoptosis. Taken together, our data identify a novel PYCR2/MASTL/Wnt/β-catenin signaling pathway that promotes colon cancer.

## 5. Conclusions

Overall, in this current study, we establish the specific role of PYCR2, out of the other PYCR enzymes, as a critical regulator of cellular proline homeostasis in colon cancer. Our results, along with two other recent independent studies [33,55], provide rigorous support for PYCR2 having a causal role in CRC. We also show that PYCR2 impacts the CSC population by regulating cancer cell survival in a MASTL/Wnt-signaling-dependent manner. These novel findings will help pave the way for future determinations of PYCR2/MASTL/Wnt/β-catenin signaling as a novel biomarker of CRC aggressiveness and as a potential therapeutic target.

## Data Availability

The data presented in this study are available in this article and supplementary material.

## Supplementary Materials

The following supporting information can be downloaded at https://www.mdpi.com/article/10.3390/cells12141883/s1: Figure S1: PYCR2 expressions in CRC significantly associated with poor patient survival. Figure S2: Effects of PYCR2 loss of expression in CRC cells using different genetic manipulation tools. Figure S3: PYCR2 regulates the intracellular proline level. Figure S4: Effects of transient (siRNA-mediated) and constitutive (shRNA plasmid) PYCR2-KD on markers of apoptosis/cell death in HCT116 and SW620 cells. Figure S5: PYCR2 modulates MASTL/Wnt/β-catenin signaling. Supplementary Table S1: List of siRNAs, shRNA, CRISPR/Cas9, pcDNA3 PYCR2, and MASTL cDNA clones used in this study. Supplementary Table S2: List of antibodies and reagents with their dilutions used in our study. Supplementary Table S3: List of RT-qPCR primers used in this study.
