# Supplementary material for: Pyrroline-5-Carboxylate Reductase-2 Promotes Colorectal Carcinogenesis by Modulating Microtubule-Associated Serine/Threonine Kinase-like/Wnt/β-Catenin Signaling
Source: Cells. 2023 Jul 18;12(14):1883. doi: 10.3390/cells12141883 (PMC10377831; doi:10.3390/cells12141883)
Supplement: Supplementary file 1 [file cells-12-01883-s001.zip › cells-2452379-supplementary.pdf]

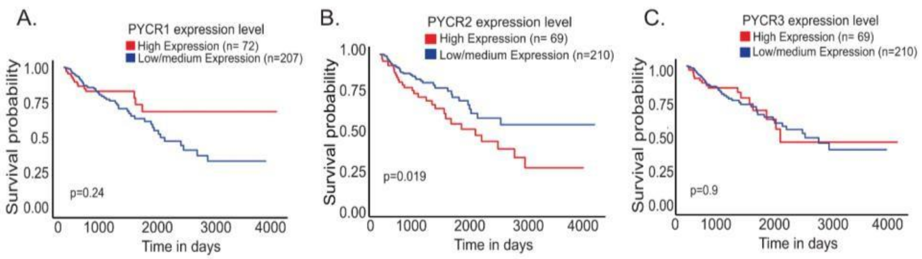

Figure S1. PYCR2 expression in CRC significantly associated with poor patient survival. (A) The Kaplan-Meier (Km) plot survival probability analysis for PYCR1 in CRC patients (*p*=0.24). **(B)** The Km plot analysis for survival probability of CRC patient with PYCR2 expression *(p*=0.019*).* **(C)** The Km plot analysis for survival probability of CRC patient with PYCR3 expression (*p*=0.9).

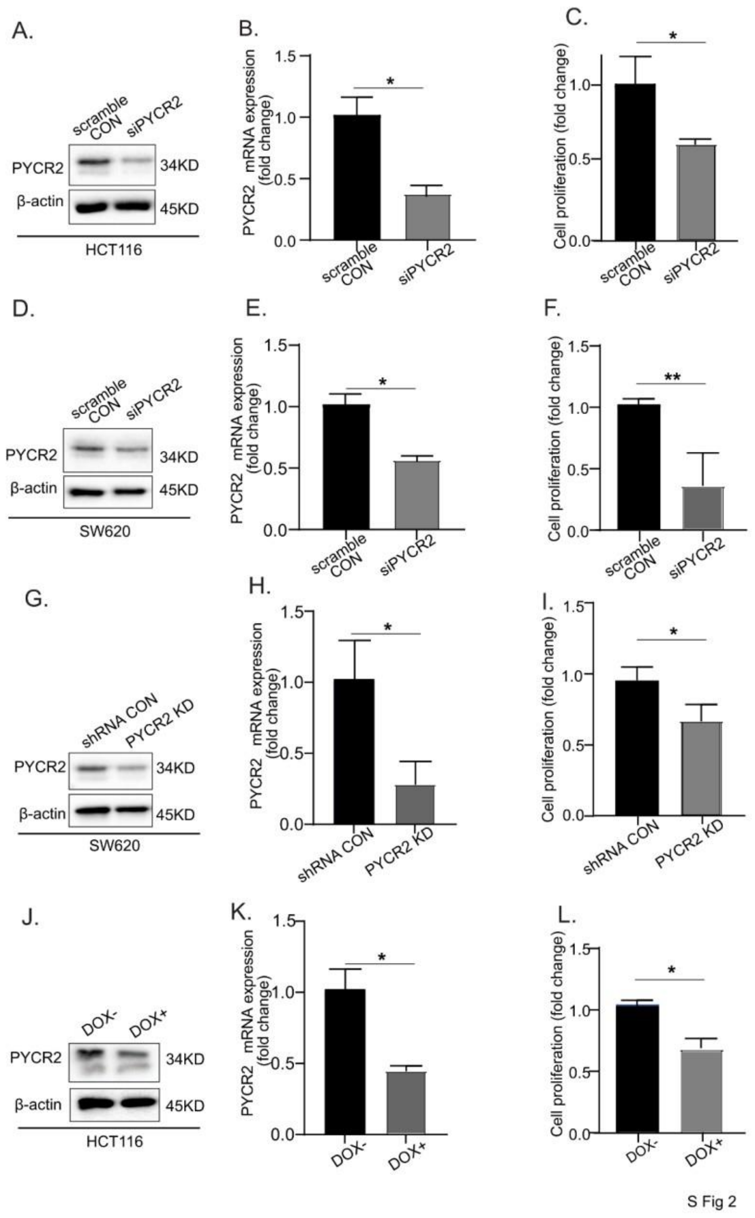

Figure S2. Effects of PYCR2 loss-of-expression in CRC cells using different genetic manipulation tools. (A-F) The loss of function of PYCR2 was performed by using the anti-Human PYCR2 siRNA in HCT116 and SW620 cells. Representatives

immunoblot, and RT-qPCR analysis for PYCR2 mRNA for HCT116, and SW620 respectively and cell proliferation assay for HCT116 and SW620 cells respectively. **(G-I)** PYCR2 knockdown using constitutive shRNA plasmid in SW620 cells. Representative immunoblots of PYCR2 mRNA expression and cell proliferation analysis. **(J-L)** PYCR2 knock down using doxycycline inducible shRNA plasmid in HCT116 cells. The representative images of immunoblots, PYCR2 mRNA expression analysis and cell proliferation assay. *p<0.05, **p<0.01.

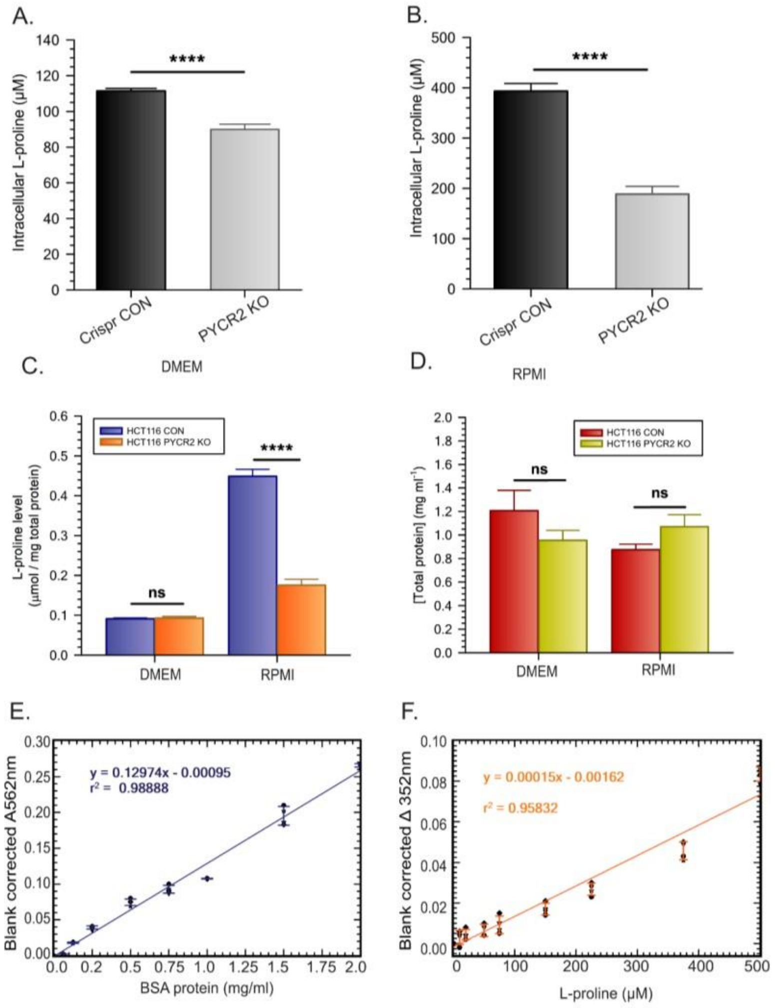

Figure S3. PYCR2 regulates the intracellular proline level. Intracellular L-proline concentrations in HCT116 control and PYCR2-KO (knock out) cells cultured in DMEM. **(A)** and in RPMI-1640. **(B)**. Quantification was performed using modified acid ninhydrin assay**. (C)** Normalized intracellular L-proline levels in HCT116 cells cultured in different growth media and collected at 10^5 cells. Data plotted as mean ± SEM of (n=3) biological replicates, each in triplicate. L-proline concentration values in (μM) units measured by using modified acid ninhydrin assay, were normalized to corresponding total protein concentration values in (mg ml^{-1}) units measured by using BCA Pierce protein assay, to express vertical axis as intracellular L-proline level in (μmol/mg total protein) units. **(D)** Total protein concentrations in HCT116 cells cultured in different growth media and quantified by using BCA Pierce protein assay. Data plotted as mean + SEM of (n=3) biological replicates, each in triplicate. **(E)** BSA known protein concentration standards' calibration plot using BCA Pierce protein assay. **(F)** L-proline known concentration standards' calibration plot using modified acid ninhydrin assay. Statistical significance was determined by Student's t-test, ****, $p < 0.0001$, ns, not statistically significant.

$$\mathrm{L-proline\ level\ (\mu mol/mg\,total\ protein)} = \frac{\mathrm{L-proline\ concentration\ (\mu mol\ L^{-1})}}{\mathrm{Total\ protein\ concentration\ (mg\ mL^{-1})}} \times \frac{\mathrm{1\ L}}{\mathrm{10^3\ mL}}$$

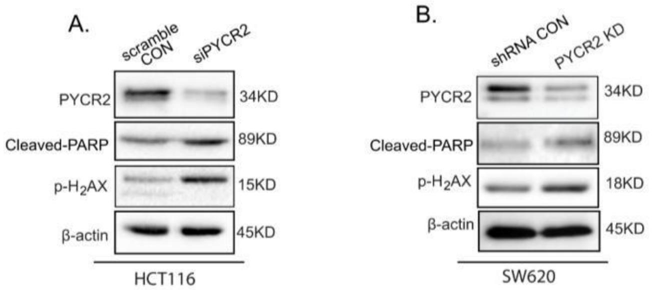

Figure S4. Effects of transient (siRNA-mediated) and constitutive (shRNA plasmid) PYCR2-KD upon markers of apoptosis/cell death in HCT116 and SW620 cells. (A-B) The immunoblot analysis for $p-H_2AX$ and cleaved PARP expression after PYCR2 knock down.

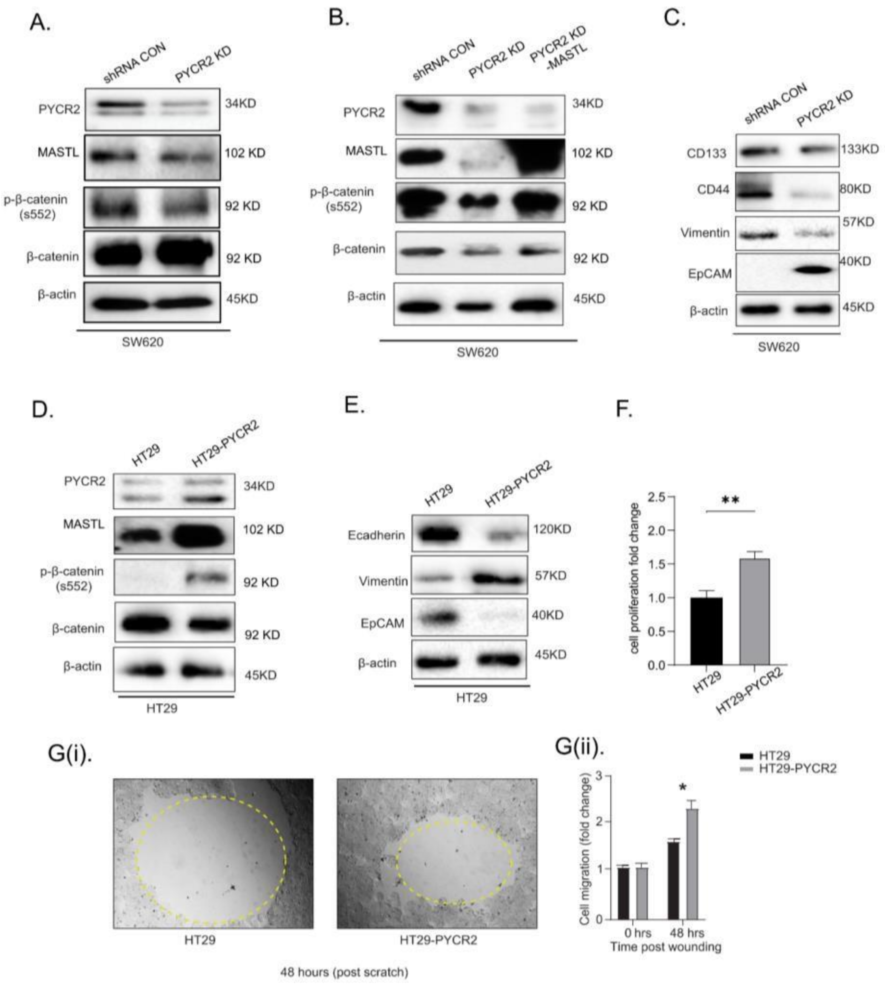

Figure S5. PYCR2 modulates the MASTL/Wnt/β-catenin-signaling. (A-B) Representative immunoblot analysis to investigate the expression of MASTL/Wnt/β-catenin-signaling in control, PYCR2 KD and MASTL overexpression in SW620 cells. **(C)** Immunoblot analysis for CSCs and EMT markers in PYCR2-KD SW620 cells. **(D-E)** Immunoblot analysis of MASTL/Wnt/β-catenin-signaling along with EMT markers in HT29-PYCR2 cells. **(F-Gi-ii)** Cell proliferation and cell migration assay in control and HT29-PYCR2 cells. **p*<0.05, ***p* < 0.01

Table **S1. List of siRNA, shRNA, Crispr/Cas9, pCDNA3 PYCR2 and MASTL cDNA clone used in study**

Name	Company	Catalogue	Sequence
Human Anti-PYCR2 siRNA	Ambion™	AM16708	Sense: GGUCCUUGUGAUAAAACCUtt<br>Antisense: AGGUUUUAUCACAAGGACCtg
Human Anti-PYCR2 shRNA	Sigma Millipore	TRCN0000046368	GCCCTTAAGAAGACCCTCTTA
SMARTvector™ Inducible Human PYCR2 shRNA	Dharmacon	V3SH11252-226257373	AGCAGCGTACGCAGGGTTG
3× sgRNA/Cas9 all-in-one expression clone targeting PYCR2	Genecopoeia	HCP262887-CG12-3-B	a)<br>atccGTGAACCGCGGACCATGAGCGGTTTTAGAGCTAGAAATAGCAAGTTAAAATAAGGCTAGTCCGTTATCAACTTGAAAAAGTGGCACCGAGTCGGTGCTTTTTT<br>b)<br>atccGAGCTGGCCTATGCTCTGGCGGTTTTAGAGCTAGAAATAGCAAGTTAAAATAAGGCTAGTCCGTTATCAACTTGAAAAAGTGGCACCGAGTCGGTGCTTTTTT<br>c)<br>atccGAAGCCCCGCGCCAGAGCATGTTTTAGAGCTAGAAATAGCAAGTTAAAATAAGGCTAGTCCGTTATCAACTTGAAAAAGTGGCACCGAGTCGGTGCTTTTTT
MASTL PCS6 (pCMVSPORT6)	Transomic	BC009107	
pCDNA3 PYCR2			Prepared in lab

Table S2. List of antibodies and reagents with their dilution used in the study.

SN	Name of Antibodies/chemicals	Company	Catalog #	Dilution used
1	PYCR2	ProteinTech	17146-1-AP	1:1000 (WB) and 1:250 (IHC)
2	β-actin	ProteinTech	20536-1-AP	1:20000
3	p-H2AX	Cell Signaling	2577	1:1000 (WB) and 1:500 (IHC)
4	Cleaved PARP	Cell Signaling	5625S	1:1000 (WB)
5	p-AKT	Cell Signaling	4060	1:1000 (WB)
6	AKT	Santa Cruz	Sc-5298	1:1000 (WB)
7	Cyclin D1	ABclonal	A19038	1:1000 (WB)
8	Cleaved caspase 3	Cell Signaling	9664S	1:1000 (WB) and 1:1000 (IHC)
9	Mastl	Sigma Millipore	MABT372	1:1000 (WB)
10	p-β-catenin	Cell Signaling	5651S	1:1000 (WB)
11	β-catenin	BD Bioscience	610154	1:1000 (WB)
12	EpCam	Cell Signaling	3599S	1:500 (IF)/1:1000 (WB)
13	Ecadherin	BD Bioscience	610405	1:1000 (WB)
14	Vimentin	Cell Signaling	5741S	1:1000 (WB)
15	CD133	Cell Signaling	64326T	1:1000 (WB)
16	CD44	Cell Signaling	3578S	1:1000 (WB)
17	SOX2	Cell Signaling	2748S	1:1000 (WB)
18	Precision plus protein standard	Biorad	1610375	
19	GKI			

Spheroid media protocol (50ml)

- B-27 supplement (# 17504044 Thermofisher) -1X
- HEPES (# 15630106 Thermofisher) - 10mM
- DMEM/F-12, GlutaMAX (# 10565018 Thermofisher)- Remaining volume

Table S3. List of qPCR primers used in study.

PYCR1

1	Forward	GGACAAGGTGAAGCTGGACT
	Reverse	AGGACGTGTCAATCCTTGC
2	Forward	TTTCTGCTCTCAGGAAGATG
	Reverse	ACCACAATGTGTCTGTCCTC
3	Forward	AAGATGGCAGGCTTGTGGAGCA
	Reverse	CAGAGCATCCAGGGCTGTGAAA

PYCR2

1	Forward	TCCCTCGCTGAGGGGGTTCGT
	Reverse	CCATCTTCCTGAGCGCGGACACC
2	Forward	AGATGGGTGTGAACCTGACA
	Reverse	CTTCACAGCCAGAAACAGGA
3	Forward	CGGCTCACAAGATAATAGCC
	Reverse	TTCACCGTCTCCTTGTTGC

PYCR3

1	Forward	CCCAGACCCTGCTGGGGGACG
	Reverse	CTCCACGGCGCTCATGGTGG
2	Forward	GTGGAAGCTCAGCACATACTGG
	Reverse	CTTGGTGGCAAAGATGACGAGC

CD133

Forward	GGTGCTGTTCATGTTCTCGA
Reverse	ACCGACTGAGACCCAACATC

CD44

Forward	CCAGAAGGAACAGTGGTTTGGC
Reverse	ACTGTCCTCTGGGCTTGGTGTT

Sox2

Forward	GAGCTTTGCAGGAAGTTTGC
Reverse	GCAAGAAGCCTCTCCTTGAA

β-actin

Forward	CACCATTGGCAATGAGCGGTTC
Reverse	AGGTCTTTGCGGATGTCCACGT
